# Does telehealth influence the decision to transfer residents of residential aged care facilities to emergency departments? A scoping review

**DOI:** 10.1111/opn.12517

**Published:** 2022-11-17

**Authors:** Carla Sunner, Michelle Therese Giles, Ashley Kable, Maralyn Foureur

**Affiliations:** ^1^ Nursing and Midwifery Research Centre, Hunter New England Area Health Service Newcastle New South Wales Australia; ^2^ School of Nursing and Midwifery University of Newcastle University Drive Callaghan New South Wales Australia

**Keywords:** care homes, care of older people, decision‐making, emergency Department, gerontological Nursing, long‐term care, residential care, telehealth, transfer

## Abstract

**Background:**

Emergency Departments (ED) can be crowded places and not ideal environments for Residential Aged Care Facilities (RACF) residents awaiting assessment. Assessment and care planning may be made available via telehealth thereby avoiding unnecessary transfer to ED, without compromising the quality of care for the older person. Telehealth is attractive addition to improving healthcare decision‐making in RACFs.

**Objectives:**

The aim of this scoping review is to explore the evidence around the use of telehealth and whether it influences the decision to transfer residents of RACF to ED.

**Methods:**

All peer reviewed literature that focused on RACFs, decision‐making and assessment of residents using telehealth in real time, was included. All study designs, pilot studies and some systematic reviews were considered. Databases Medline, Embase and CINAHL were used in this search in June 2022. Search terms were a combination of the population: RACF residents, decision‐making and assessments using telehealth, and or transfer to the ED. The search was assisted by a senior university research academic librarian/information specialist and reviewed by senior researchers. The PRISMA‐ScR guidelines were used to report this study.

**Results:**

Of the 124 articles initially identified, 31 were eligible for inclusion for synthesis. The date range of the included studies was 2001 to 2022, with 15 published in the last five years. Critical appraisal was conducted using the Mixed Methods Appraisal Tool.

**Conclusion:**

This scoping review has mapped evidence that telehealth has been widely used in multiple settings. The association between the use of telehealth with improved clinical outcomes highlights its potential utility in enhancing care delivery for an older population in RACFs. Telehealth has shown that it can improve the decision‐making for residents in RACFS, but more robust research designs are needed.

**Implications for practice:**

Using video/telehealth appears to improve RACF staff access to expert clinicians who can then assess and jointly plan care/management that can be provided in the resident's home. Knowledge and skills of RACF staff appear to be improved through joint assessment and decision‐making with the use of video/telehealth access to expert clinicians.

## 1 INTRODUCTION

Hospitalisation of residential aged care facility (RACF) residents can potentially affect their quality of life, expose them to unnecessary health risks and increase their mortality and morbidity. Emergency departments (ED) can be crowded places and not ideal environments for residents awaiting assessment. With prolonged wait times and length of stays (LOS) in ED, residents are at risk of iatrogenic complications and adverse events (Grant et al., 2020). It would be beneficial for RACF residents to have access to viable alternatives to ED thereby avoiding unnecessary health risks. Alongside the risk of being in ED, is the fact that the resident's visit may have been unnecessary, as found in studies in the United States and Canada, where 67% and 25% (respectively) of RACF‐resident presentations were potentially avoidable (Gillespie et al., 2019; Grant et al., 2020).

Alternative care pathways and health management plans for RACF residents rather than an unnecessary and risky hospital visit is worthy of consideration. Cost‐effective strategies like telehealth are an attractive addition to improving the provision of healthcare within RACFs (Chan et al., 2001). Telehealth may support the RACF staff to make better decisions about the care required and available care options. There is evidence that adding a visual assessment to a telephone consultation can improve the quality of care and clinical decision‐making (Jarvis‐Selinger et al., 2008) for RACF residents. The Internet boom has led to the expansion of telehealth/telemedicine applications available for use globally (WHO, 2009), which has made telehealth technology more accessible.

## 2 BACKGROUND

There are many descriptions of telehealth that identify it as mobile and dynamic communication tool, adaptable to the medical needs of the care recipient in many ways and across many contexts. Telehealth and telemedicine are terms that are often used interchangeably. For the purpose of this paper, we will refer to any interaction that uses real‐time visual assessment consultation as telehealth, including telemedicine. They are both performed in real time involving synchronous data transmission. Telemedicine is often used to describe occasions where there is a physician or health professional who is assessing and prescribing treatment via the use of telehealth (WHO, 2009). Telehealth fits with the definition from the International Organisation for Standardisation that defines telehealth as the use of telecommunication techniques for the purpose of providing telemedicine, medical education and health education over a distance (ISO, 2021).

Telehealth provides a way of reducing inequalities in health care by delivering knowledge, resources and skills to support staff in rural communities where they do not have ready access to clinical expertise (Nesbitt, 2012). Teleradiology is an example of a critically important acute care telehealth service provided to rural hospitals to assist in the rapid diagnosis of patients with traumatic injuries and strokes. Telestroke is a model telehealth service because of its documented improvements in patient outcomes and the strong economic case that can be made for implementing the service (Weinstein et al., 2014).

Teletrauma, teleburn and telestroke programs (Weinstein et al., 2014) bring capability and urgent assessment and treatment to areas that do not have such specific clinical expertise. In ophthalmology and optometry, non‐mydriatic cameras can be used to perform retinal screenings in people with diabetes without needing to dilate the eyes, and this has increased screening rates. Correctional telehealth contributes to not having to transport prisoners to outside clinics and protecting public safety (Weinstein et al., 2014). Examples of telehealth include, but are not limited to, telepaediatrics, telecardiology, teledermatology, teleinfectious disease, teleneurology, teleophthalmology, telepathology, telepulmonology, telepsychiatry, telerheumatology and telenursing (Weinstein et al., 2014).

In recent years, there has been a rapid uptake of technology internationally. This has intensified the scope and availability of telehealth, utilising Web‐based applications (e.g. e‐mail, teleconsultations and conferences via the Internet) and multimedia approaches (e.g. digital imagery and video) (WHO, 2009). Staff and patient satisfaction have increased in the past few years, probably due to familiarity with, and improvements to technology (Weiner et al., 2001). Despite these technological advances, the use of telehealth has not progressed as rapidly as expected.

Despite the availability of ready to use telehealth devices in industrialised and developing countries (Wootton, 2008), the process of activating telehealth conversations is not straightforward (Weinstein et al., 2014). Reasons for this are reported to be the lack of standards (Nesbitt, 2012), poor progress once initial ‘seed’ money dries up (Wootton, 2008), lack of financial incentives and poor technology integration (Weinstein et al., 2014). There is also the problem of competing workload commitments for the staff in RACFS caring for residents with higher health needs who require extensive support (Gillespie et al., 2019). Furthermore, it is challenging to maintain the clinical skills needed for RACF staff when there are high staff turnover rates (Gillespie et al., 2019), this is a valid concern for implementation planning. In addition, negative staff attitudes towards telehealth can have an impact on telehealth implementation (Crundall‐Goode & Goode, 2014).

This paper presents a scoping review of the literature to identify evidence of the effectiveness and experience of telehealth use in RACFs to assist the decision‐making of RACF staff regarding the transfer of the resident to ED. Limited literature seems to be available describing or evaluating how a telehealth model of care can prevent RACF residents from presenting to EDs. A scoping review of the literature will identify what information is available in all clinical areas to understand the barriers and enablers to telehealth, and if telehealth improves the clinical decision‐making, and how an intervention can be implemented successfully into practice.

A scoping review is considered less restrictive than a systematic review with search criteria allowing a broader scope for literature searches (Munn et al., 2018). In addition, in a scoping review, information can be drawn from any source and is not restricted to quantitative studies (Munn et al., 2018). In the case of RACF‐resident transfers to ED, a scoping review will be invaluable in identifying existing evidence (Munn et al., 2018) and help the reader understand the key concepts and concerns of an approach rather than describe the efficacy and viability of interventions that a systematic review provides. The scoping review potentially provides an overview or a map of the evidence and clarification of definitions (Munn et al., 2018).

### 2.1 Aim

The aim of this scoping review is to explore if the use of telehealth and whether it influences the decision to transfer residential aged care facilities residents to emergency departments.

## 3 METHODS

Our protocol was developed using the scoping review methodological framework proposed by Arksey and O'Malley (2005) and further refined by the Joanna Briggs Institute (JBI) (Peters et al., 2020). A scoping review of the literature as outlined by Arksey and O'Malley (2005) was undertaken to examine the extent, range and nature of the research undertaken in this area, to assist in summarising the research findings, and identify gaps in the existing literature. As Peters et al. (2020) describe, scoping reviews differ from other reviews in that, they are used to present a broad overview of the evidence, regardless of the quality of the study and are considered a precursor to a systematic review. They are useful to uncover emerging data, clarifying key concepts and identifying gaps (Peters et al., 2020). The Scoping review was completed in six stages as recommended by (Arksey & O'Malley, 2005) 1. Identifying the research question; 2. Identifying relevant studies; 3. Study selection; 4. Charting the data; 5. Collating, summarising and reporting the results; and 6. Consultation (with information specialist librarian). This study followed the Preferred Reporting Items for Systematic reviews and Meta‐analysis Extension for Scoping Reviews (PRISMA‐ScR) guidelines (Tricco et al., 2018) see File S1 and the ENTREQ Statement (Enhancing the transparency in reporting the synthesis of qualitative research) File S2.

### 3.1 Search strategy

The search strategy for this scoping review was developed with assistance from a university research librarian/information specialist, following the JBI framework to determine eligibility of the search question (Peters et al., 2020) including studies that assessed;

1. The population: RACF residents (65 years or older),
2. Concept: decision‐making and assessments using telehealth,
3. Context: transfer to the ED.

The full electronic search strategy was refined in the Medline database, including any limits used, such that it could be repeated and is presented in File S1. These search terms were also used in subsequent searches of databases, Embase and CINAHL, with all papers fitting search criteria to July 2022. All relevant articles retrieved from this search strategy were included for screening. Additional studies were identified by manually searching the reference lists of potentially relevant papers and other telehealth/telemedicine systematic reviews. All articles identified were imported into Covidence Systematic Review Software for screening File S3.

### 3.2 Eligibility criteria

#### 3.2.1 Inclusion criteria

All peer reviewed literature that focused on RACFs, decision‐making and assessment of residents using telehealth in real time were included. All study deigns using recognised methods of data collection and data analysis including, some pilot studies and some systematic reviews were considered, and only studies reporting evidence relating to RACFs were included. Studies were included if they were in English language only.

#### 3.2.2 Exclusion criteria

Articles not pertaining to telehealth, decision‐making or integrating patient care in real time with telehealth were not included, along with discussion papers/editorials or papers which only have abstracts available.

### 3.3 Information sources

#### 3.3.1 Title and abstract

Title and abstract screening was completed in Covidence Systematic Review Software. The inclusion criterion was followed as above, for the title and abstract screening. Review articles, conference abstracts, posters, editorials and commentaries were excluded from the review.

#### 3.3.2 Full text screening

Full text screening was also completed in Covidence Systematic Review Software. Papers included were based on blinded review by two authors using the following four main concepts:

1. Telehealth, telemedicine (visual/video/real‐time);

2. Nursing homes, long‐term care homes, RACFs or alternatives;

3. Decision‐making and/or assessment;

4. Emergency service and/or alternative.

When the screening was completed by two independent reviewers, the conflicts were managed and resolved by discussion between the two reviewers, including a third reviewer when necessary.

### 3.4 Data extraction and analysis

A data extraction form on Microsoft Excel was used to guide the collection of information from each article. The following descriptive data were extracted from each article that satisfied the inclusion criteria: year of publication, language, country, study design and study setting. For completed studies (not protocols), participant demographics were extracted, including number of participants, and participant age and sex. With respect to telehealth, we extracted information about persons who undertook telehealth consultations, and assessment. All outcomes and variables that studies assessed for a telehealth relationship with RACF residents, assessment and emergency were recorded. As numerous outcome variables were identified, outcomes were grouped according to overarching themes for the purpose of analysis.

## 4 RESULTS

**FIGURE 1:**
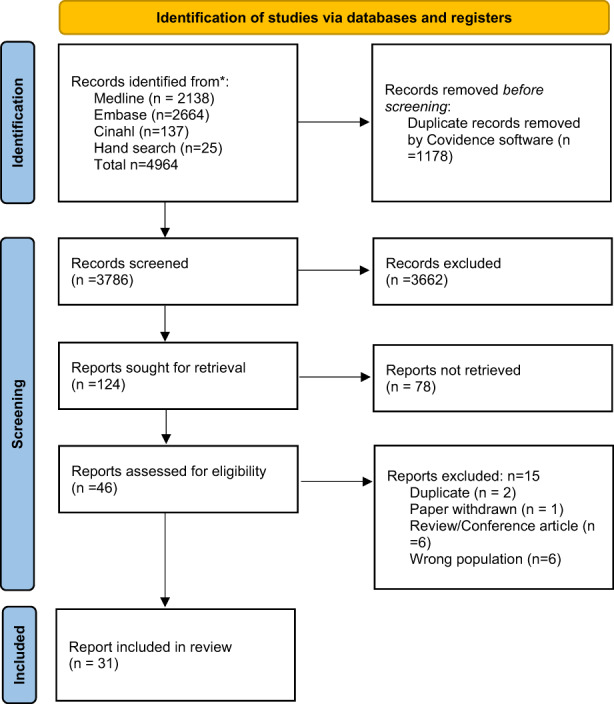
Prisma flowchart (Page et al., 2020)

Our database search retrieved 4939 articles and hand‐searching identified 25 additional articles for a total of 4964. After duplicates were removed, articles were included for title and abstract screening. We screened the full text of 124 articles these were further assessed till we were able to retrieve 31 papers for this scoping review that fitted our criteria. The findings from this search process are presented in Figure 1.

### 4.1 Critical appraisal of individual sources of evidence

The quality of the reported studies was appraised using the mixed methods appraisal tool (MMAT) (Hong et al., 2018). The rationale for using the MMAT tool was that it was able to assist appraisal across different study designs and methodologies using only one tool. The MMAT tool was created and validated to assess the methodological quality of five categories of studies: qualitative research, randomised controlled trials, non‐randomised studies, quantitative descriptive studies and mixed method studies (Hong et al., 2018). Whereas it is not mandatory to utilise a tool for critical appraisal in a scoping review, the authors felt that the inclusion of the MMAT would provide rigour, reduce bias and a uniformed approach towards appraisal of the studies found.

The MMAT (Hong et al., 2018) measures overall quality, with two initial questions where further appraisal may not be needed if the answer is no to one or both screening questions. All the papers were critically appraised by the first author and divided up evenly between the other authors of this paper, so all studies were appraised by two people independently. Any discrepancies were discussed until a consensus was reached. Six of the papers were identified as poor in quality (scoring 4 or below) but were still included in the Evidence Table (Table 2).

### 4.2 Data charting process

An overview of each included paper is provided in Table 1. This evidence table provides further details of the authors, year of publication, country, context, aim, study design, sample data collection methods and analysis, outcomes/ findings, MMAT score and limitations.

**TABLE 1:** Evidence table describing studies included in the scoping review

Authors date country	Context	Aim	Study design	Sample	Data collection methods and analysis	Outcomes/findings	MMAT score	Limitations
RCT								
Grabowski & O′ Malley, 2014, USA	NH* a mix of post‐acute and long‐stay residents	To determine whether residents of NH that were randomly chosen to receive off‐hours physician coverage by a telemedicine service experience a lower rate of hospitalisation, compared to residents of homes that received standard physician coverage.	RCT	11 NH (6 control 5 intervention)	NH demographics, hospital transfers, monthly data number and reasons for telehealth calls.	Reduction in hospitalisations: control group 5.3%: intervention group 9.7%, however, the difference was not statistically significant for this telemedicine intervention. However, when the researchers compared more (−8.08) and less‐engaged treatment facilities (0.36), a significant decline in the hospitalisation rate was observed at more‐engaged facilities (difference = −8.40), (for a nursing home that would have had 180 hospitalisations per year without the use of the telemedicine service. p < 0:05) A NH that typically had 180 hospitalisations per year and that was more engaged with telemedicine would have a reduction of about 15.1 resident hospitalisations each year	Fair	Data to track long stay or short stay residents missing.
Joseph et al., 2020, USA	Residents from 6 urban SNF*.	To determine whether a SNF* based telemedicine consultation service staffed by Emergency Physicians (EP*) will reduce hospital admissions, compared to residents taken directly to ED	RCT	4606 residents 2311 intervention 2295 control.	Using electronic health records data about telemedicine, ambulance transfers, odds ratio of hospital admissions.	Only 27% (*n* = 624) of the residents from intervention group SNF were transported to the ED, compared to 71% (*n* = 1629) of the control (OR = 0.15 [95% CI, 0.13–0.17], *p* < .001)	Fair.	Seasonality was a potential confounder
Cluster randomised stepped wedge trial								
Kane‐Gill et al., 2021, USA	4 × NHs (2 urban, 2 suburban)	To determine the impact of pharmacist‐led telemedicine services on reducing adverse drug reaction (ADE*)	Cluster randomised stepped wedge trial.	984 ED admissions reviewed	Incidence of high‐risk medication, alert‐specific ADEs and all‐cause hospitalisation including 30‐day readmission rates	The intervention group had a 92% lower incidence of alert‐specific ADEs than usual care (9 vs 31; 0.14 vs 0.61/1000‐resident‐days; adjusted incident rate ratio = 0.08 (95% confidence interval (CI) = 0.01–0.40]; P = 0.002)	Good	Even though ADE categories were exhaustive miscategorised cases occurred
Stern et al., 2014, Canada	12 eligible LTC* facilities	To determine the cost effectiveness of enhanced multidisciplinary teams via telemedicine for the treatment of pressure ulcers	Cluster randomised stepped wedge trial	12 LTCs were randomly allocated a start date to link LTC residents to a hospital‐based multidisciplinary wound care team via phone, email or telehealth	Outcomes for 137 residents with pressure ulcers reviewed In‐depth interviews Economic evaluation	Primary outcome rate in reduction of pressure ulcers and secondary outcome was ED visits, wound healing times and hospitalisations. No statistically significant differences were found between control and intervention periods on any of the primary or secondary outcomes	Good.	Hard to embed the intervention due to frequent staff turnover and insufficient managerial attention
Pre–post								
Hullick et al., 2022, Australia	RACFs in a metropolitan setting	To determine whether adding video telehealth consultation to established ACE (Aged care emergency outreach) program further reduces ED visits and hospital admissions	Quantitative non‐randomised pre–post study. 14 months of pre‐data compared with 14 months of post‐data	5 intervention RACFs were compared with 8 control RACFs	Patient clinical and demographic characteristics, hospital admission and ED visits, admission status, admission diagnosis, data relating to ED visits	There were no significant differences in hospital admission or ED visits after the introduction of video‐telehealth; adjusted incident rate ratios (IRR) were 0.98 (95% CI 0.55 to 1.77) and 0.89 (95% CI 0.53 to 1.47) respectively.	Good	Investigating the impact of a single intervention as part of a larger multicomponent intervention is difficult and may require different methodologies for evaluation
Mixed methods								
Hui & Woo, 2002, Hong Kong	1x 200‐bed NH with telemedicine	To determine the feasibility, acceptability and cost‐effectiveness of telemedicine provided geriatric services to residents of nursing homes	Mixed methods	1001 teleconsultations were made and with 876 survey participants including Geriatricians, Psycho‐geriatricians, dermatologists, Nurses, Physiotherapists, Occupational therapists, podiatrists	Survey and cost analysis	96% residents favoured the service, and they felt comfortable with this mode of consultation and its convenience. Lower operating costs than for conventional services. Shorter wait times for referrals. Increase of confidence with nursing staff in caring for residents	Fair	Physical examination limitations
Laflamme et al., 2005, USA.	Nursing home residents (NHR) urban	To pilot and assess the role of videoconferencing in clinicians' medical decision‐making and their interactions with nursing home residents (NHRs).	Mixed methods study. Paired virtual and face‐to‐face (FTF) bedside examinations with the FTF examination immediately following the videoconference—by the same clinician	NHRs (*n* = 35) and clinicians (*n* = 3) receiving or providing routine care between 2002 and 2003.	Clinician–NHR interactions were assessed using coding review of videos with a 31‐item instrument. Clinician rating of videoconference. Clinician orders categorised and counted.	For 71% of the encounters, clinicians stated that videoconferencing facilitated their assessment. Difficulties included sound quality (19%) and participants' familiarity with videoconferencing (7%). Although NHRs were alert in 50% of encounters, 62% of alert NHRs did not indicate understanding of the recommended treatment	Good	Small sample size. May not be representative of other practitioners in similar settings. Randomisation would have limited the sample size available in each group
Piau et al., 2018, France	10 × LTC	To assess the LTC staff perception of telemedicine	Descriptive	Numbers not provided for staff. Included in the study were nurses, psychologists, physicians, GP	Pre and post intervention telehealth semi‐structured interviews with LTC staff. Questionnaires and demographic information of residents	Staff had positive perceptions of telemedicine. Strengths, weaknesses, threats (SWOT) analysis reported that potential threats became weakness, there was a fear of dehumanising medicine. 180 telemedicine sessions over 2 years with LTC staff	Good	LTC facility staff reported that it was difficult to engage with GPs
Salles et al., 2017, France	39 × NHs	To describe 1. The implementation of the interactive telemedicine in NHs. 2. The geriatric assessment performed during telemedicine consultation	Mixed method descriptive study	Audit of 500 telemedicine consults relating to 304 residents. Survey of NH participants numbers not reported	Data audit; demographics, including mean age, ADL* and MMSE* score rate, characteristics of telemedicine calls. Satisfaction survey of NH team	Avoided transfer to hospital in 378 (75.6%) cases [specialised consultations (*n* = 264, 52.8%); programmed hospitalisations (day hospital and hospitalisation, *n* = 110, 22.0%), and emergency admission (*n* = 4, 0.8%)]. Inappropriate prescriptions were corrected in 351 cases (70.2%). The NH teams reported 92% satisfaction with the intervention	Fair	No tools were used to quantify NH staff improvement. Results concerning skills improvement of the NH staff were based on an impression and needed to be explored further
Weiner et al., 2001, USA	240 bed urban nursing home	To explore the use and efficacy of rapid video conferencing of acute medical problems in the nursing home residents	Mixed methods	27 physicians and 187/369 residents randomised to have video calls, only 50% could complete the questionnaire, due to cognitive deficit	Physician rating score and a Patient Questionnaire	15/27 physician ratings of successful video recordings, with 54% satisfaction with video communication, the medical decision was easier 83%, with 87% video calls attended from home totalling 394 calls	Poor	
Descriptive								
Corcoran et al., 2003, Hong Kong	200‐bed home for the elderly	To investigate the acceptability of using telemedicine in the diagnosis of foot disorders	Descriptive	49 residents surveyed, 1 = podiatrist	Survey	Podiatrist found in almost 80% of cases telemedicine to be acceptable; 87% of residents preferred teleconsultations as opposed to being transported to the hospital, with 99 podiatry teleconsultations	Good	Only one podiatrist available to participate in the study
Driessen, 2016, USA	907 nursing home (NH) provider physicians (present at a conference)	To determine the perceptions of the potential effectiveness of telemedicine in RACF‐resident assessments	Descriptive cross‐sectional	907 were invited and 435 surveys completed by providers, physicians, a 49.5% response rate	Survey	Outcome—Among NH providers, there is a high degree of confidence in the potential for a telemedicine solution impacting upon potential avoidable hospitalisations in NHs; did not feel as though telemedicine would reduce resident care effectiveness, poorly utilised telemedicine service in NH	Good	Self‐selected study sample, small sample size, not generalisable because it is a purposive sample
Gray et al., 2012, Australia	LTC 441 bed	To determine the nature and volume of telehealth services that might be provided to a LTC	Descriptive	402 residents (132 low care, 254 high‐care, 16 special care)	Data analysis, demographics, consult specialty, location, reason for consult, transport method to hospital	146 medical consultations out of a total of 744 led to an emergency or unplanned hospital admission. The 598 (18%) consultations (excluding emergency hospital visits and GP consultations) related to 23 medical specialities	Good	External consultations were not included so numbers may be underestimated
Harris et al., 2021, USA	48 residents of a post‐acute/LTC	To determine whether collaboratively utilised telehealth centred strategies can improve residents' outcomes in a pandemic	Descriptive	13 residents treated with the use of telehealth	Data collected; demographics hospitalisation rates, reason for telehealth consult, outcomes, deaths, data analysis = descriptive statistics/frequencies	Telehealth = A handheld multifunction examination platform that integrates videoconferencing with a stethoscope, otoscope and an oral camera along with Bluetooth‐enabled vital sign monitoring. 1) rapid identification of residents who required escalation of care, (2) standardisation of care monitoring for residents who remain in the facility (3) care coordination to facilitate. Bidirectional transfers between the facility and the hospital, (4) clarification of goals of care for palliation rather than acute care transfer, and (5) daily facility needs assessment	Fair	
Low et al., 2020, Singapore	8 × NHs = 1600 beds, 1x general hospital, 1x geriatric medical department	To determine the clinical profile of teleconsultations conducted between an acute geriatric medical department and NHs	Prospective Descriptive	1673 teleconsultations 850 unique residents between 2010 and 2017	Resident profiles, Demographics, presenting diagnosis, management and process provided by the teleconsultation	The 4 most common chronic patient conditions seen via teleconsultations were hypertension (57.2%, *n* = 957), dementia (40.4%, *n* = 676), diabetes (38.7%, *n* = 647) and hyperlipidaemia (38.2%, *n* = 639). Within a month after the consultation, 83.6%, *n* = 1399 of the consultations had the residents remaining in the NH for continuing care, whereas 3.4% *n* = 57 passed away in the NH	Good	The outcome measures and quality indicators were self‐reported by the NHs
Ohligs et al., 2020, Germany	1 NH, and a General practitioner (GP)	To describe the holistic tele‐medical system for a NH which facilitates the connection to a GP and avoids unnecessary hospitalisation. To determine acceptability of the model	Descriptive	56 routine and emergency teleconsultations	Structured Interviews with; GPs, a subset of residents, nurses and nursing management	Model helpful and even necessary for careful and reliable decision‐making by the GP; show high acceptance in retirement homes. Involved residents, nurses and the general practitioner itemise various specific benefits including economic, personal and altruistic issues	Fair	No economic consequence of how telemedicine could save the health dollar
Pallawala & Lun, 2001, Singapore	2 × elderly homes	To determine the acceptability of implementing telegeriatric medical service with the use of technology to remote sites where the elderly homes are based	Descriptive	Not reported	Interviews with nursing staff and residents around useability and resident management	Nursing staff feel that there are reduced the transfers (no data provided), significantly improved management of residents, increased confidence in an event of emergency. Residents preferred teleconsultation as transfer leads to many physical problems and ailments, confident, perceived the system as a valuable resource that offered great potential	Poor	Numbers of survey participants not reported in study
Prandi et al., 2020, Italy	1 × NH residents	To determine risks, benefits, effectiveness, efficiency and future adoptions of telemedicine to address the management of malnourished residents. Using eViSuS (VV*) a telecare remote video‐visit system	Descriptive	10 residents	Survey of residents/caregivers from NH who were visited through VV	Primary outcome was if resident satisfaction was achieved, and users would employ VV again. 100% felt comfortable with telehealth doctor's medical skill, 90% fine with being on camera, 80% felt that were private. VV was as good as face‐to‐face visit (agreement 70%, neutral 30%) and it was easy for residents to state their concerns during VV (agreement 80%, neutral 20%)	Poor	Small number of completed surveys and the caregivers were not always present. Small study
Rabinowitz et al., 2010, USA	NH residents/family and RACF staff	To describe the development and implementation of a nursing home telepsychiatry consultation service and the benefits associated with its use	Descriptive	106 nursing home residents; 278 telepsychiatry encounters	Cost and time savings analysis compared to usual care. Encounter and patient, NH, charge, provider travel costs, characteristics	The telepsychiatry approach had a cost savings of 843.5 hours of travel time saved, decrease in 26.4 workdays. For the treatment of 106 residents	Fair	Not generalisable, with only a single hospital experience
Tynan et al., 2018, Australia	4 × RACFs, 1 regional, 3 rural	To describe the development and implementation of an oral health integrated telehealth model of care and outline the lessons learnt	Descriptive study using ethno‐methodology approach	33/116 residents required teledentistry	Pre‐ and post‐chart audits—number of appointments avoided at a facility, cost estimates, field notes about decision‐making process	Pre‐implementation of telehealth; 53% of residents at participating RACFs had an oral healthcare plan Post‐implementation; increase to 96%. *N* = 116, tele dentistry 33 (28%), required appointments at a facility 19 (16%), avoiding facility presentation 97 (84%)	Fair	Obtaining consent from families for oral health review by the oral health team for eligible residents was difficult
Wakefield et al., 2004	Two Veterans Affairs Medical Centres (VAMC) and a state Long‐term care (LTC) centre	To assess provider and resident satisfaction with and outcomes of specialist physician consultations provided via interactive video to residents of a long‐term care (LTC) centre	Quantitative descriptive using a cross‐sectional Survey	Physicians (*n* = 12) at the VAMC. Nurses (*n* = 30) and residents (*n* = 62) at the LTC centre	Satisfaction ratings and record review to determine changes in treatment plan and follow‐up care	*N* = 76 individual consultations in six clinics. Treatment plan with the resident remaining at the LTC setting (*n* = 29, 38%) or no change in treatment (*n* = 26, 34%). Physicians' ratings of the interactive video as good to excellent for usefulness in developing a diagnosis =78%, usefulness in developing a treatment pla*n* = 87%, for quality of transmissio *n* = 79%, and satisfaction with the consult format = 86%. Overall, 72% of residents were satisfied with the consult format, and 92% felt that it was easier to obtain medical care via telemedicine. Nurses felt it was good use of their time and skills (100%)	Good	All participants were male so the sample is not representative of the target population which includes females, some residents were unable to participate due to cognitive impairment which excluded them from the study
Retrospective cohort studies								
Hex et al., 2015, UK	27 care homes with telemedicine 968 beds 21 care homes without telemedicine as a control 557 beds	To determine acute hospital activity before and after installation of telemedicine in care homes	Non‐randomised retrospective comparison study	942 residents from telemedicine group, 502 residents from the non‐telemedicine group	Data audit; hospital admissions and ED visits were gathered for care home residents who had used telemedicine a costing for each ED attendance was £114.56	With telemedicine, emergency admissions declined by more than 1700 compared to the same time period prior. Cost analysis return on investment in telehealth would result in 39% cost saving re LoS* reduction in the acute hospital, a cost reduction of £5.23 million compared to the same period before	Fair	The availability and the quality of the community after hours services
Roques & Hovanec, 2001, USA	1x 60 bed Assisted living facility (*ALF)/ a 66‐bed licensed SNF/social workers	To determine whether a telemedicine program helped link residents with psychiatric symptoms in the ALF and SNF to the clinical teams at the major hospital	Cohort study, pre–post implementation of telehealth	126 resident transfers	Audit of number of residents transferred to hospital and length of stay 1 year after implementation	Comparing 1998 to 1999, there were ‘Fairly dramatic’ changes in the number of hospitalisations 50% decline (*n* = 67) and a total 100 fewer days spent in hospital	Poor	Set up of equipment could be seen as a prohibitive expense
Chess et al., 2018, USA	Skilled nursing facility (SNF)	To determine the clinical and financial impacts of using telehealth with an after‐hours physician	Cohort study	259 residents out of 313 bed (SNF), convenience sample	Audit of resident conditions/diagnosis, financial impact data‐hospital avoidance, ambulance transfers, LoS	Cost savings; total number of telehealth calls were 313 with 259 (83%) residents were treated on site, including 91 who avoided hospitalisations, 54 were transferred to the hospital. Estimated cost savings to Medicaid and other payers exceeded $1.55 million; improving nurse assessment skills; comfort and confidence to families; and resident integration within the clinical team	Good	Only in one facility in one context, not generalisable
Grounded theory design								
Stephens et al., 2020, USA	1 × NH	To explore the care givers experiences/challenges with NH to ED transfers and whether telehealth might be able to mitigate some of those concerns	Grounded theory	41 participants comprising of; families, NH nurses, primary care providers, ED physicians, ED nurses, NH administrators	Focus groups	ED were perceived as having a lack of trust in NH care and capabilities. Four main factors: questioning the quality of NH nurses' assessments, perceptions that physicians were absent from the NH; misunderstandings of the capabilities of NHs and EDs; and perceptions. That responses to medical needs were inadequate	Good	There was an inability to engage NH residents in the focus groups, missing the resident's. perspective of the intervention
Case study								
Bidmead et al., 2015, UK	5 Nursing homes (NH) and their speech and language therapists (SLT*)	To build a case for adoption of the ‘teleswallowing’ assessment model, explore barriers and enablers	Case study; action research methodology	SLTs = 6, Managers = 5, Nurses = 3, Residents = 17	2 focus groups (SLTs), Interviews (7 telephone, 5 Face‐to‐face)	Upskilled staff in nursing homes; quicker assessments/shorter waiting times; avoidance of serious problems and hospital admission; less distress for residents and improved quality of life; benefits to residents and nursing homes from not attending hospital outpatient appointments; prestige for participating nursing homes; and freeing up SLTs' time	Good	Technical problems, staffing pressures, in sufficiently experienced SLTs. Improvements wanted; iPad are heavy, using a camera would be better, dedicated IT support, protocol, and criteria development
Pilot study/Proof of concept / feasibility								
Archbald‐Pannone et al., 2020, USA	Long‐term care (LTC) facilities	To support optimal care of residents during COVID‐19 with the use of telemedicine and strengthening all communication links	Pilot study	12 LTC contacted to use telemedicine only 5 participated	Collected information on the number of telemedicine calls	Strong evidence does not yet exist regarding efficacy or effectiveness of telemedicine in LTC facilities found in this project. The mortality rates from the LTC COVID‐19 outbreaks were 12% and 19% lower in participating LTCs compared to the published mortality rate of 28% (presumed of the general LTC population, not clear)	Poor	Numbers were not clear from data, only % provided
Catic et al., 2014, USA	LTC and large academic hospital with strong geriatric medicine program	To design, implement and assess the pilot phase of an innovative, remote case‐based video consultation program called Extension for Community Healthcare. Outcomes (ECHO‐AGE). A multidisciplinary group providing assessments via videoconferencing platforms	Pilot study	11 LTC 47 participants used ECHO‐age; data analysed for 44 participants only	Audit of outcome and hospitalisations	ECHO‐AGE recommendations followed for 39 out of 44 residents in the long‐term care sites reported that 74% (*n* = 29) of the residents clinically improved (*p* < .03 2‐sided Fisher exact test). In addition, hospitalisation was less common among 29% (*n* = 11) of residents in whom the recommendations were followed	Poor	Agitation was not defined well enough. Low numbers
Chan et al., 2001, Hong Kong	1 Nursing home (NH) with 198 residents over 12 months	The feasibility and acceptability of using tele‐nursing in a NH Intervention—using telemedicine to assess residents in 7 key health areas	Descriptive	Surveys, 47 residents, 18 NH staff, 9 Geriatric outreach team members	Survey of useability and acceptability of telemedicine. Hospital transfer data	Potentially economically attractive with the reduction in travel and nurse escorting residents to hospital. 89% (*n* = 176) assessments could be done as telemedicine and treated insitu where 11% (*n* = 23) were sent to ED. Survey data 96% residents considered teleconferencing acceptability was good. Developed strong partnerships between NH and hospital, whilst increasing staff confidence	Fair	One facility only. Impractical equipment size of telemedicine workstation. Dependant on the cooperation of NH staff
Hofmeyer et al., 2016, USA	34 LTCs	To determine whether the use of telemedicine to assess residents from rural LTCs will reduce potentially avoidable hospitalisations (PAH*) to hospital	Descriptive proof of concept	23 eLTC* calls per implemented LTC per year, over 3 years	Data analysis; number of eLTC consults, transfers to ED, number of potentially avoidable hospitalisations	69% (*n* = 511) did not require a transfer to ED. Prior to the implementation of eLTC they would most likely have needed transfer to ED	Fair	Staff of the eLTC felt that they were contacted too late to be able to resolve issues when called by the LTC
Hui et al., 2001, Hong Kong	1 × 200 bed NH	To assess whether providing telegeriatric services to NH residents result in an increase in productivity and saving	Mixed methods, pilot study	876 survey participants, 356 patient episodes of care Geriatric assessment team, specialists, NH staff and residents	Survey, cost analysis	Deemed a cheaper alternative. Attitudes of nursing staff was generally positive, however, half felt that their workload increased after telemedicine was installed, 30% of residents could not complete the survey due to dementia diagnosis ED attendance reduced from 328 to 299 visits per annum. There were 1001 telemedicine consults	Fair	

Abbreviations: ACE, aged care emergency; ADE, adverse drug reaction; ADL, activities of daily living; ALF, assisted living facility; ED, emergency department; EP, emergency physicians; FTF, face to face; eLTC, electronic long‐term care; GP, general practitioner; LoS, length of stay; LTC, long‐term care; NH, nursing home; NHR, nursing home residents; MMSE, mini mental state examination; PAH, preventable hospital admissions; RACF, residential aged care facility; SLT, speech language therapist; SNF, skilled nursing facility; VAMC Veterans Affairs Medical Centres; VV eViSuS telecare remote video‐visit system.

### 4.3 Synthesis of results

#### 4.3.1 Description of studies

**Authors** of the 124 articles identified for full text review, 31 were eligible for inclusion in our synthesis and are presented in the Evidence Table describing studies included in the scoping review (Table 1).

**Date,** the date range of the studies was 2001 to 2022, with 15 published in the last 5 years.

**Country,** the various countries represented in this review are described in the Evidence Table (Table 1). Most studies were from the United States (*n* = 15) followed by Hong Kong (*n* = 4) and Australia (*n* = 3), two each from Singapore, France and the UK, and one each from Germany, Italy and Canada.

**Context,** for the purpose of this review, we use the range of terms found in the studies where RACFs are referred to as; elderly homes, nursing homes, skilled nursing facility (SNF), long‐term care and assisted living facility. The RACFs were located across a mix of urban and rural environments. The studies were led by different discipline groups, including pharmacists (Kane‐Gill et al., 2021), General Practitioners (GP) (Ohligs et al., 2020; Weiner et al., 2001), specialist physicians (Wakefield et al., 2004) geriatricians (Catic et al., 2017; Driessen et al., 2016; Pallawala & Lun, 2001), podiatrists (Corcoran et al., 2003), psychiatrists (Rabinowitz et al., 2010), social workers (Roques & Hovanec, 2002), dentists (Tynan et al., 2018), speech pathologists (Bidmead et al., 2015) and hospital departments and RACFs (Archbald‐Pannone et al., 2020; Chan et al., 2001; Chess et al., 2018; Grabowski & O'Malley, 2014; Gray et al., 2012; Harris et al., 2021; Hex et al., 2015; Hofmeyer et al., 2016; Hui et al., 2001; Hui & Woo, 2002; Hullick et al., 2022; Joseph et al., 2020; Laflamme et al., 2005; Low et al., 2020; Piau et al., 2018; Prandi et al., 2020; Salles et al., 2017; Stephens et al., 2019; Stern et al., 2014).

**Aim,** the studies aimed to determine if telehealth; lowered rates of hospitalisations, reduced adverse events, was cost effective, was an acceptable service, improved health outcomes, linked people to appropriate services, was an effective and efficient intervention, and strengthened communication links. Further to this, studies also aimed to understand; clinical staff perception of telehealth, what it was being used for, the benefits, barriers and enablers, how much activity was going on, and the care givers' experiences.

**Study Design,** two studies were cluster randomised stepped wedge trials (Kane‐Gill et al., 2021; Stern et al., 2014), and two were randomised controlled trials (Grabowski & O'Malley, 2014; Joseph et al., 2020) a pre/post‐study (Hullick et al., 2022). Four studies used mixed methods (Hui & Woo, 2002; Laflamme et al., 2005; Piau et al., 2018; Salles et al., 2017), and 39% of studies were descriptive (*n* = 12) (Corcoran et al., 2003; Driessen et al., 2016; Gray et al., 2012; Harris et al., 2021; Low et al., 2020; Ohligs et al., 2020; Pallawala & Lun, 2001; Prandi et al., 2020, Rabinowitz et al., 2010; Tynan et al., 2018; Wakefield et al., 2004; Weiner et al., 2001). Three studies were retrospective cohort studies (Chess et al., 2018; Hex et al., 2015; Roques & Hovanec, 2002), one used a grounded theory study design (Stephens et al., 2020) and one a case study (Bidmead et al., 2015). Five were pilot studies (Archbald‐Pannone et al., 2020; Catic et al., 2017; Hui et al., 2001), including a proof‐of‐concept study (Hofmeyer et al., 2016).

**Sample,** the sample sizes varied from describing numbers of RACF residents (*n* = 4606) (Joseph et al., 2020) or number of RACFs (*n* = 11) (Grabowski & O'Malley, 2014) or admission numbers to ED from RACFs (e.g. *n* = 984) (Kane‐Gill et al., 2021), or number of teleconsultations made (*n* = 1001) (Hui & Woo, 2002).

**Data collection methods and analysis, a** range of data collection methods were used, including surveys, interviews, medical record audits and cost analyses. A range of statistical and qualitative data analysis techniques were used according to the type of data collected.

**Outcomes/Findings,** RACFs that were more engaged with telehealth had significant reductions in hospitalisations per year (Grabowski & O'Malley, 2014; Hofmeyer et al., 2016; Salles et al., 2014), less adverse events (Kane‐Gill et al., 2021), lower operating costs and increased staff satisfaction (Hui & Woo, 2002; Piau et al., 2018), found it acceptable (Corcoran et al., 2003; Wakefield et al., 2004), improved coordination of care and improved outcomes (Harris et al., 2020; Laflamme et al., 2005), high acceptance by GPs and nurses (Ohligs et al., 2020) and increased nurse confidence (Pallawala & Lun, 2001), acceptable for the residents (Prandi et al., 2020), significant cost‐savings and operating costs (Chan et al., 2001; Chess et al., 2018; Hex et al., 2015; Rabinowitz et al., 2010). However, some studies found that telehealth did not result in a significant reduction in ED visits (Hullick et al., 2022; Stern et al., 2014), was poorly utilised with no perceived confidence in its ability to influence hospital avoidance (Driessen et al., 2016), and that there were misunderstandings surrounding the telehealth and clinical abilities of the staff in the RACFs (Stephens et al., 2019).

**MMAT score,** Critical appraisal using the MMAT (Hong et al., 2018) resulted in 13 of the studies receiving a rating of good, 12 were rated as fair, and 6 were rated as poor due to low methodological quality. The data are presented in Table 2.

**TABLE 2:** MMAT study rating

	MMAT score range	Number	Studies
Good	5–7	13	Bidmead et al., 2015; Chess et al., 2018; Corcoran et al., 2003; Driessen et al., 2016; Gray et al., 2012; Hullick et al., 2022; Kane‐Gill et al., 2021; Laflamme et al., 2005; Low et al., 2020; Piau et al., 2018; Stephens et al., 2020; Stern et al., 2014; Wakefield et al., 2004.
Fair	4–5	12	Chan et al., 2001; Grabowski & O'Malley, 2014; Harris et al., 2021; Hex et al., 2015; Hofmeyer et al., 2016; Hui & Woo, 2002; Hui et al., 2001; Joseph et al., 2020; Ohligs et al., 2020; Rabinowitz et al., 2010; Salles et al., 2017; Tynan et al., 2018
Poor	0–4	6	Archbald‐Pannone et al., 2020; Catic et al., 2017, Pallawala & Lun, 2001; Prandi et al., 2020; Roques & Hovanec, 2002; Weiner et al., 2001

**Limitations,** of some studies included that seasonality was a confounder for hospital presentations, poor coding, and data collection issues, difficult to embed the intervention due to staff turnover, physical examination limitations, difficulties engaging with GPs, small sample sizes meaning the study findings were not generalisable, and some studies made claims of reductions in events without providing any numerical or statistical data.

### 4.4 Findings

For the qualitative analysis, two authors conducted the initial categorisation of the key components independently, using NVivo (QSR, 2020) and manually, then presenting the results to the team for discussion. A framework was established through team discussions upon reviewing the preliminary results as a guide, as recommended by JBI. Categories were then identified, coded and charted using significant text from the papers, using the framework as a guide. The qualitative evidence and quantitative evidence were brought together in an overarching synthesis and in a final iteration and consensus by all authors.

Analysis of the 31 studies, identified five common findings: 1. Older person (resident) hospital avoidance, 2. Older person (resident) experience, 3. Nurses' improved assessment skills, 4. Cost savings, and 5. Barriers and enablers. The five findings are presented in the following section.

### 4.5 Older person (resident) hospital avoidance

Most of the papers identified increases in hospital avoidance as an outcome measure. However, only two studies used robust research designs with low risk of bias, including an RCT (Joseph et al., 2020) and a Stepped wedge RCT (Stern et al., 2014). These two trials reported conflicting results. The Joseph et al. (2020) RCT found that the telehealth groups were less likely to have their care escalated to a hospital than the control groups that has no telehealth service 27% vs 71% (OR 0.15, CI 0.13‐0.17); whereas the Stern et al. (2014) Stepped Wedge RCT did not find a significant difference in hospitalisation rate and it was estimated to be 1.2 (CI 0.62, 2.36) times more(*p* = .59) with the use of telehealth.

The Hofmeyer et al. (2016) pilot study found that 69% of 511 telehealth consults could be managed without an ED transfer. In a descriptive study by Low et al., (2020), keeping the resident in the RACF after the telehealth interaction was perceived to be a successful outcome;

‘Within a month after the consultation, 83.6% of 1399 consultations had the patients (sic) remaining in the nursing home’ (Low et al., 2020, p. 1075).

In other studies, a small pilot study found that hospitalisation was less common in 29% (*n* = 11) of residents in whom the telehealth recommendations were followed (Catic et al., 2017), and in a mixed method study, 75.6% (*n* = 378) avoided transfer to hospital (Salles et al., 2017). In a case study examining identification of residents with urgent podiatry problems (Corcoran et al., 2003), there was earlier identification and avoidance of serious problems with the use of telehealth that would otherwise have necessitated a hospital admission (Bidmead et al., 2015). In a descriptive study that measured just the physicians' impressions, it was reported that they had confidence that telehealth would impact on hospital avoidance (Driessen et al., 2016; Laflamme et al., 2005). This finding was also confirmed in a retrospective cohort study by Roques and Hovanec (2002, p. p 37). The authors claimed that

‘During the first year of operation of the Tele‐medicine program, there were fairly dramatic changes in the number of hospitalizations (1997 n=21, 1998 n=11) and the total number of days spent in the hospital (1997 n=367, 1998 n=258)’ (Roques & Hovanec, 2002, p. 37).

#### 4.5.1 Older persons' (resident) experience

Seven of the included studies reported on the residents' experience of telehealth. Pilot studies (Chan et al., 2001; Hui et al., 2001) considered the resident experience to be positive and acceptable. Resident satisfaction was measured through surveys and interviews, all reporting a positive experience in terms of usability and acceptability of telehealth (Chan et al., 2001). A case study interviewed residents about telehealth. Residents indicated that they felt less distress and increased comfort and they felt it gave them a better quality of life with the use of telehealth (Bidmead et al., 2015). Importantly, a cohort study found that residents also felt more included in decision‐making with telehealth (Chess et al., 2018). Further, a descriptive study utilising a survey found that residents regarded communication via telehealth made it easy to state their concerns (Prandi et al., 2020).

Important insights were provided in a descriptive study (Corcoran et al., 2003), that reported a preference for telehealth over person‐to‐person appointments;

‘87% (n=40/46) preferred teleconsultations to being transported to the hospital clinic for their foot care’ (Corcoran et al., 2003, p. 148).

Two studies, a case study, and a pilot study, commented on how hard it was to recruit enough participants for surveys due to the frequency of a dementia diagnosis among residents (Bidmead et al., 2015; Hui et al., 2001). Hui et al., (2001) reported that 70% of residents were unable to provide informed consent to participate in a survey due to cognitive impairment (Hui et al., 2001). This was a common reason given for low numbers of residents' perspectives included in many studies.

#### 4.5.2 Nurses' improved assessment skills

Telehealth offered RACF staff the opportunity for a second opinion and supported teaching/learning when a more skilled colleague or clinician was involved in the consultation. A grounded theory study by Stephens et al. (2020) explored the effect of telehealth on the assessment skills and experience of the RACF nurse with the use of telehealth.

‘The staff of the residential home found that it increased their knowledge and ability to care for the client. Initially, some were anxious about telemedicine, but after a little experience they became confident and adept at using the equipment’. (Corcoran et al., 2003, p. 148).

This in turn, built trust with the residents because their care was being managed in collaboration with many clinicians (Stephens et al., 2020). Other studies have described telehealth in RACFs as improving staff confidence due to ‘Improving nurse assessment skills’ (Chess et al., 2018, p. 386; Hui & Woo, 2002; Pallawala & Lun, 2001), increasing professional satisfaction (Catic et al., 2017) and assisting in the avoidance of adverse events (Bidmead et al., 2015).

Whilst seven studies reported on the improvement of assessment skills for staff, only one study questioned whether these skills would be sufficient to assist the recipient on the end of the telehealth call. There were concerns from speech language therapists (SLT) regarding accountability associated with making the correct diagnosis for the resident via telehealth (Bidmead et al., 2015). One SLT commented

‘would we get enough information from the person at the other end of the link to allow us to give safe recommendations?’ (Bidmead et al., 2015, p. 6).

#### 4.5.3 Cost savings

The use of telehealth in cost analyses focused on the reduced travel of the resident to ED and the cost of the specialist travel to the resident (Corcoran et al., 2003; Rabinowitz et al., 2010). In some cases, the additional cost of sending an escort with the resident to ED was saved (Gray et al., 2012), along with the cost of the ambulance transfer. Reported cost savings varied considerably from hundreds of dollars to some resulting in million‐dollar savings (Chess et al., 2018; Grabowski & O'Malley, 2014; Hofmeyer et al., 2016), depending on the RACF and the study size. A study by Chess et al. (2018, p. 386) stated there are ‘significant healthcare cost savings’. This study found in one year, 91 residents avoided hospital admission, ambulance transfers and Medicaid‐covered costs, with a saving of US$1.6 million with the inclusion of a telehealth enabled service. Two studies (Chan et al., 2001; Grabowski & O'Malley, 2014) reported that telehealth was economically attractive, with a study by Hex et al. (2015) reporting a 39% return on investment in the reduction of length of stay in hospital. In a stepped wedge trial, telehealth made an indirect care cost reduction in wound care of US$650 per resident compared to usual care (Stern et al., 2014).

#### 4.5.4 Barriers and enablers

##### 4.5.4.1 Enablers

All studies found telehealth to be very acceptable. Studies that included older people (residents), podiatrists, nurses, occupational therapists, psychiatrists, dentists, GPs and geriatricians all found telehealth to be of benefit for several reasons. Reasons included the development of a good working relationship between the GP, nurse and the resident (Ohligs et al., 2020); the GP was able to pass from one resident to the next in almost no time; allowing an efficient use of resources, and telehealth was understood to be an efficient triage mechanism that identified issues in timely fashion (Corcoran et al., 2003; Harris et al., 2021). Podiatrists also found that telehealth allowed earlier intervention for residents and nursing staff who increased their knowledge and ability to care for the resident using visual telehealth (Corcoran et al., 2003).

Telehealth allowed integration within the clinical team and the primary attending physician (Chess et al., 2018); a great benefit to the clinical assessment process. This benefit was realised in that the physician can see and examine the resident within minutes and can initiate treatment or send the resident to hospital in a timelier way (Chess et al., 2018). An efficiency also noted by speech therapists and podiatrists, telehealth allowed them to see more residents (Bidmead et al., 2015; Corcoran et al., 2003). A podiatrist study reported increased productivity;

‘Three times the number of people could be screened via teleconference in the same amount of time as required for an on‐site consultation’. (Corcoran et al., 2003, p. 148).

In addition, specialists reported that the use of telehealth helped to reduce waitlist assessments for residents (Corcoran et al., 2003; Hui & Woo, 2002).

##### 4.5.4.2 Barriers

Two studies indicated that staff felt their workload had increased with the introduction of telehealth (Bidmead et al., 2015; Hui et al., 2001). Along with the perception of increased workload, it was hard to embed the intervention due to staffing issues such as insufficiently experienced staff (Bidmead et al., 2015). The staffing of caregivers in RACFs was hampered by frequent staff turnover and insufficient managerial support (Stern et al., 2014). In addition to the poor staffing, there were issues around provider engagement with telehealth as there was not always a guarantee that RACF staff would use telehealth when offered it. This poor uptake of telehealth was reported by Grabowski and O'Malley (2014) in two out of the six RACFS in their study. Furthermore, Hofmeyer et al. (2016) emphasised that if the physician is contacted about a deteriorating resident by the RACF staff too late, the impact of telehealth is diminished.

One study in France reported that there was a difficulty engaging GPs with telehealth (Piau et al., 2018). The GP is the primary health provider for residents and their involvement was not reported in many of the studies in this review. Of the 28 studies, there were only three that mentioned GP involvement specifically (Ohligs et al., 2020; Piau et al., 2018; Salles et al., 2017) and not always positively. One study implementing a telehealth pharmacological intervention reported that some GPs;

‘refused to implement the proposed pharmacological interventions, which was very frustrating for the staff’ (Piau et al., 2018, p. 1002).

Several authors identified telehealth was not always appropriate for physical examinations (Corcoran et al., 2003; Hui et al., 2001; Piau et al., 2018) or procedures like the debridement of a wound (Hui & Woo, 2002; Piau et al., 2018) or other ‘hands on’ procedures (Corcoran et al., 2003, p. 148). This was clarified in the Corcoran et al. (2003) podiatry study, with authors concurring that telehealth would not always be appropriate because:

‘assessment via teleconference did not include in‐depth neurological or vascular assessment, because of the lack of equipment on site’. (Corcoran et al., 2003, p. 149).

Similarly, geriatricians found it limiting to do a physical examination on new residents and nurses found it was challenging to assess resident behavioural problems (Hui & Woo, 2002). In contrast, a survey of providers revealed that telehealth would reduce avoidable hospitalisation of residents and not weaken their care management plan (Driessen et al., 2016).

## 5 DISCUSSION

Few RCTs have been conducted that report a treatment effect associated with a telehealth intervention (Grabowski & O'Malley, 2014; Joseph et al., 2020; Kane‐Gill et al., 2021; Stern et al., 2014). The stepped wedge trial by Kane‐Gill et al. (2021), a pharmacist‐led patient‐centred telehealth project, had a 92% lower incidence of adverse drug reactions compared to usual care because of the telehealth consultation and review of medications (*p* = .002, 95% CI = 0.01–0.4). The Grabowski and O'Malley (2014) RCT identified that if a RACF experiencing an average of 180 hospitalisations per year engaged with telehealth, they could expect to see a statistically significant reduction of about 15.1 hospitalisations each year, compared to RACFs that were less engaged. The Stern et al. (2014) stepped wedge trial supported the finding that quality of leadership and high staff turnover in RACFs impacted the uptake of telehealth. Their qualitative data analysis revealed that the use of wound care consultations were highly feasible in hospital avoidance. The Stern et al. (2014) study estimated that mean hospitalisation rates were 1.2 times higher during the intervention due to a more thorough wound care evaluation (95% CI 0.62–2.36), although this was not statistically significant (p‐.59). In the Joseph et al. (2020), RCT‐skilled nursing facilities using telehealth had significantly less ED transfer rates (27%, *n* = 637) when compared to the control group (71%, *n* = 1629), (*p* < .0001). All trials were conducted in the United States or Canada.

The review of these studies identified the following issues. Firstly, there was an absence of detail about the actual lived experience of a telehealth consultation and the perspectives of RACF residents (Stephens et al., 2020). Corcoran et al. (2003) reported that residents were often excluded due to a diagnosis of dementia. Additionally, there was no measurement identified regarding the impact of the seasonal variation on hospital presentations. Only one study acknowledged that there may have been a seasonal confounder (Joseph et al., 2020). Finally, there was an issue in some cases around data accuracy. One study reported there was a problem capturing accurate information with the incorrect categorisation of telehealth cases (Kane‐Gill et al., 2021) and missing numbers in another study (Gray et al., 2012).

No studies have examined the effectiveness of an ED outreach service plus telehealth capability in reducing ED presentations from RACFs. There is no evidence that we can locate of such a model having been trialled and no previous studies have reported the effectiveness of such an integrated approach. Studies about the use of telehealth and hospital avoidance are mainly based around increasing access to care from different types of clinicians, for example, wound care, podiatry, geriatricians (Catic et al., 2017; Driessen et al., 2016; Pallawala & Lun, 2001) and speech pathology (Bidmead et al., 2015). Furthermore, in many studies, the sample sizes were small with the sample often drawn from only one RACF, and consequently, results were not generalisable (Chess et al., 2018).

Supportive collaborations with clinical staff and RACF staff using telehealth to enhance access to teaching and learning, knowledge, and skill development, can increase the ability for staff to care for the RACF resident (Corcoran et al., 2003). Telehealth in one study has shown that these collaborations resulted in 69% of consults preventing an ED transfer (Hofmeyer et al., 2016) and hospitalisation was less common with telehealth with 122 cases (24.4%) (Salles et al., 2017).

Not only can telehealth be justified as a way for residents to avoid an unnecessary hospital admission, it has additional patient‐centred benefits also. Studies identify high levels of staff satisfaction and confidence with the use of Telehealth (Chess et al., 2018; Hui & Woo, 2002; Pallawala & Lun, 2001). Studies also reported a positive experience for residents in terms of usability and acceptability of telehealth (Chan et al., 2001), feeling less distress (Bidmead et al., 2015) and having increased comfort. Another positive outcome was that the resident and families were involved in the decision‐making surrounding the management of their care. Residents also felt more included in the decision‐making with telehealth (Chess et al., 2018) which they felt gave them a better quality of life (Bidmead et al., 2015). In accessing telehealth, residents have an opportunity to be involved in decision‐making and a choice in their own health care justifying the use in RACF care pathways.

Hospital avoidance was observed in most studies yet telehealth is not commonly embedded in all acute facilities. Telehealth is not used to its full potential and was viewed in one descriptive study to be underutilised (Driessen et al., 2016). Grabowski and O'Malley's (2014) RCT suggested the reason for telehealth underutilisation may be the need for policy reform to incentivise the use of telehealth with financial remuneration. The cost savings from engaging with telehealth and return on investment has been mentioned in many of the studies. Telehealth is a justifiable investment for healthcare services with the appropriate and cost‐effective care pathways for RACF residents.

There is a need to conduct research related to RACF residents to help prevent unnecessary hospital admissions and readmissions. Telehealth outreach models can further support RACF staff to care for residents in RACFs with the opportunity for enhanced access to teaching and learning to help prevent unnecessary hospital presentations and treat them in the RACF without exposing them to poor outcomes in acute facilities.

Most recently, the COVID‐19 pandemic has been a catalyst for the increased use of and need for telehealth. The pandemic has unfortunately claimed more lives in the RACF‐resident cohort than in any other age bracket (Burkett et al., 2021). Clinicians are looking for ways to facilitate assessment from a distance, whilst keeping the care recipient front and centre of their care during the pandemic. The telehealth model of care (MoC) provides an effective and viable alternative for clinicians.

### 5.1 Limitations

Some studies did not recruit a representative study sample, and their results were potentially affected by some bias and consequently were not generalisable. We have added this statement below to the limitations as suggested. No a‐priori protocol was registered or published in relation to this scoping review, which is a potential limitation of the study, since a protocol aims to limit the occurrence of reporting bias. Instead, a detailed plan for the scoping review was developed with the first author and supervisory panel of academic researchers who are co‐authors. Engagement of a senior university research academic librarian/information specialist guided the search for literature aimed to ensure a sufficiently robust search was undertaken and no relevant studies were missed. Abstraction of relevant data from each paper was scrutinised by the supervisory panel as described in File S2—the use of the PRISMA‐ScR (Tricco et al., 2018) enabled the authors to check whether the scoping review conformed to this reporting standard.

## 6 CONCLUSION

This scoping review has mapped evidence that telehealth has been widely used in multiple settings. The association between the use of telehealth and improved clinical outcomes highlights its potential utility in enhancing care delivery for an older population in RACFs. Telehealth has shown that it can improve the decision‐making for residents in RACFS. Even though the studies were from a variety of different disciplines, hospital avoidance was increased. The review identified that telehealth is underutilised in RACFs. The use of telehealth in RACFs has potential for improved decisions about transferring residents and significant cost savings to hospital prior to, and during a pandemic.

**Summary statement of implications for practice**

What does this research add to existing knowledge in gerontology?

- This scoping review has mapped evidence describing the use of telehealth‐aided decision‐making in multiple settings.
- Residents are transferred out of their home when staff are unable to make confident, informed decisions about the management/treatment of the resident, in situ, in their own home.

What are the implications of this new knowledge for nursing care with older people?

- Using video/telehealth appears to improve RACF staff access to expert clinicians who can then assess and jointly plan care/management that can be provided in the resident's home.
- Knowledge and skills of RACF staff appear to be improved through joint assessment and decision‐making with the use of video/telehealth access to expert clinicians.

How could the findings be used to influence policy or practice or research or education?

- More research using robust study designs needs to be undertaken to support or refute the hypothesis that video/telehealth can improve health outcomes for residents in RACFs.
- Cost savings can be made if residents are able to receive expert care and support in their own home rather than transfer to an ED.
- Providing care for residents in their own home should become recommended government policy.

## FUNDING INFORMATION

Translational Research Grant Scheme funded project with the Ministry of Health, New South Wales, Australia (H19/53776).

## CONFLICT OF INTEREST

The authors declare no conflict of interest.

## Supporting information


Supplementary File S1

Supplementary File S2

Supplementary File S3

## Data Availability

The data that support the findings of this study are available from the corresponding author upon reasonable request.
