# Supplementary material for: Does telehealth influence the decision to transfer residents of residential aged care facilities to emergency departments? A scoping review
Source: Int J Older People Nurs. 2022 Nov 17;18(1):e12517. doi: 10.1111/opn.12517 (PMC10078385; doi:10.1111/opn.12517)
Supplement: Supplementary file 2 — Supplementary File S2 [file OPN-18-0-s001.docx]

**Supplementary File 2- ENTREQ Statement (Enhancing the transparency in reporting the synthesis of qualitative research)**

| **Item** | **Guide and description** | **Page no** |
| --- | --- | --- |
| **1. Aim** | The aim of this scoping review is to explore if the use of telehealth and whether it influences the decision to transfer Residential Aged Care Facilities residents to Emergency Departments. | 4 |
| **2. Synthesis methodology** | Our protocol was developed using the scoping review methodological framework proposed by Arksey and O'Malley (2005) and further refined by the Joanna Briggs Institute (JBI) (JBI, 2015; Peters et al., 2020). A framework was established through team discussions upon reviewing the preliminary results as a guide, as recommended by JBI (2015). | 4 |
| **3. Approach to searching** | Pre-planned, comprehensive search strategy to seek all available studies was undertaken with the authors initially with senior university research academic librarian/information specialist | 5 |
| **4. Inclusion criteria** | Phenomenon of interest: Views, experiences, and perceptions of older people in RACFs, their families and carers, and care home staff and emergency staff including physicians, nurses, allied health and GPs  Population: Older people resident in care homes, their families, and carers, and RACF staff.  Language: English language only.  Year: 2001-2022 year of publication.  Types of studies: All study deigns using recognised methods of data collection and data analysis including, some pilot studies and some systematic reviews were considered, and only studies reporting evidence relating to RACFs were included | 5 |
| **5. Data sources** | The full electronic search strategy was refined in the Medline database, including any limits used, such that it could be repeated and is presented in Supplementary File 1. These search terms were also used in subsequent searches of databases, Embase and CINAHL, with all papers fitting search criteria to July 2022. An exhaustive search of the literature was completed. | 5 |
| **6. Electronic search strategy** | Search strategy is described in detail in Supplementary File 1. | 5 |
| **7. Study screening methods** | Two reviewers (MG and CS) independently screened titles and abstracts against eligibility criteria. The full text of articles initially considered as meeting the inclusion criteria were retrieved and the eligibility criteria applied in the same way. Discrepancies at both stages were discussed and resolved with another reviewer (MF) where necessary. Categories were then identified, coded, and charted using significant text from the papers, using the framework as a guide, resulting in a qualitative content analysis. | 5-6 |
| **8. Study characteristics** | Details of the study characteristics are provided in Table 1 and a full overview of study characteristics describing studies also described. | 7-8 |
| **9. Study selection results** | Figure 1 outlines the study selection process in a PRISMA-SCR* flow diagram. | 6 |
| **10. Rationale for appraisal** | Whereas it is not mandatory to utilise a tool for critical appraisal in a scoping review the authors felt that the inclusion of the MMAT would provide rigor, reduce bias and a uniformed approach toward appraisal of the studies found. | 6 |
| **11. Appraisal items** | The quality of the reported studies was appraised using the mixed methods appraisal tool (MMAT) (Hong et al., 2018). | Table 2  6 |
| **12. Appraisal process** | The quality appraisal was conducted independently by four reviewers (MG, CS, MF & AK).  All the papers were critically appraised by CS and divided up evenly between MF, MG and AK so all papers were appraised by two people independently. Any discrepancies were discussed until a consensus was reached. | 6 |
| **13. Appraisal results** | The quality appraisal results are available in Table 2. We did not exclude any articles on the basis of quality as we believed that all studies may contribute some important insights to the phenomenon of interest. | 7 |
| **14. Data extraction** | All content in the results, discussion and conclusion sections of the included papers were considered as data for analysis. Data extraction was completed by two reviewers independently.  Information extracted: Authors, Date, Country, Context, Aim, Study Design, Sample, Data collection methods & analysis, Outcomes/ Findings, MMAT score, limitations were extracted from the included studies and are presented in Table 1. | 7-8 |
| **15. Software** | Covidence, NVivo. | 5 & 8 |
| **16. Number of reviewers** | Two reviewers (MG & CS) read all the included studies in detail and independently extracted data from the papers, meeting to discuss the findings, and reach a consensus. | 8 |
| **17. Coding** | The initial coding of the data that could not be accommodated within the framework were coded line by line to search for new themes. | 8 |
| **18. Study comparison** | The data were compared across studies and two reviewers (CS , MG) discussed. These reviewers refined the emerging concepts which included both modified and new elements that had not been anticipated in the first iteration. | 8 |
| **19. Derivation of themes** | The process of developing the themes was both deductive and inductive: initially, we identified preliminary themes and then we created new themes. | 8 |
| **20. Quotations** | Direct quotes from the papers regarding the experiences of the residents, family members and care staff - are presented in the results section of the manuscript. | 9-11 |
| **21. Synthesis outputs** | The qualitative evidence and quantitative evidence were brought together in an overarching synthesis and in a final iteration and consensus by all authors. | 8 |

*PRISMA – Preferred Reported Items for Systematic Reviews and Meta-Analyses.
