# Supplementary material for: Does telehealth influence the decision to transfer residents of residential aged care facilities to emergency departments? A scoping review
Source: Int J Older People Nurs. 2022 Nov 17;18(1):e12517. doi: 10.1111/opn.12517 (PMC10078385; doi:10.1111/opn.12517)
Supplement: Supplementary file 3 — Supplementary File S3 [file OPN-18-0-s002.docx]

Supplementary File 3 Example of MEDLINE search strategy 1946-June 2022

| **#** | **Searches** | **Results** |
| --- | --- | --- |
| 1 | (ehealth or mhealth or (e health or m health)).mp. | 16624 |
| 2 | (teleassist* or tele-assist or telebased or tele-based or teleconsult or tele-consult).mp. | 101 |
| 3 | (telecare or tele-care).mp. | 920 |
| 4 | (telediagnos* or tele-diagnos* or tele-assess or teleemerg* or tele-emerg* or telefollow* or tele-follow* or teleguidance or tele-guidance or teleintervention or tele-intervention or telemanag* or tele-manag*).mp | 459 |
| 5 | (telemed* or tele-med* or telenurs* or tele-nurs*).mp. | 44077 |
| 6 | (tele-health or telehealth).mp. | 10944 |
| 7 | (videoconferencing or video-conferencing).mp. | 4605 |
| 8 | 1 or 2 or 3 or 4 or 5 or 6 or 7 | 59495 |
| 9 | (referral and consultation).mp. | 76616 |
| 10 | Nursing Process/ | 7407 |
| 11 | (decision making or Decision Making).mp. | 255214 |
| 12 | bidirectional communication.mp. | 1057 |
| 13 | Patient Handoff/ or Interdisciplinary Communication/ | 19515 |
| 14 | (assess* or nursing Assessment).mp. | 3860246 |
| 15 | 9 or 10 or 11 or 12 or 13 or 14 | 4117205 |
| 16 | (long term care or Long-Term Care).mp. | 42003 |
| 17 | (skilled nursing facilit* or intermediate care facili*).mp. | 7617 |
| 18 | (aged or elderly or older or geriatric or ag?ing).mp. | 6265718 |
| 19 | (Residential aged care or Nursing Home*).mp. | 51992 |
| 20 | 16 or 17 or 18 or 19 | 6298177 |
| 21 | Geriatric Assessment/ or "Aged, 80 and over"/ | 1020216 |
| 22 | aged care emergency*.mp. | 7 |
| 23 | Emergency Medical Services/ or Emergency Service, Hospital/ | 125866 |
| 24 | (hospitali?ation* or hospital or emergency service*).mp. | 1578655 |
| 25 | (Emergency Medical Service* or Emergencies or Emergency Nursing).mp | 116005 |
| 26 | 21 or 22 or 23 or 24 or 25 | 2514566 |
| 27 | 8 and 15 and 20 and 26 | 2138 |
